# Epigenetic Alterations in Podocytes in Diabetic Nephropathy

**DOI:** 10.3389/fphar.2021.759299

**Published:** 2021-09-24

**Authors:** Erina Sugita, Kaori Hayashi, Akihito Hishikawa, Hiroshi Itoh

**Affiliations:** Department of Internal Medicine, School of Medicine, Keio University, Tokyo, Japan

**Keywords:** DNA damage repair, epigenetics, diabetic nephropathy, podocyte, epigenetic memory

## Abstract

Recently, epigenetic alterations have been shown to be involved in the pathogenesis of diabetes and its complications. Kidney podocytes, which are glomerular epithelial cells, are important cells that form a slit membrane—a barrier for proteinuria. Podocytes are terminally differentiated cells without cell division or replenishment abilities. Therefore, podocyte damage is suggested to be one of the key factors determining renal prognosis. Recent studies, including ours, suggest that epigenetic changes in podocytes are associated with chronic kidney disease, including diabetic nephropathy. Furthermore, the association between DNA damage repair and epigenetic changes in diabetic podocytes has been demonstrated. Detection of podocyte DNA damage and epigenetic changes using human samples, such as kidney biopsy and urine-derived cells, may be a promising strategy for estimating kidney damage and renal prognoses in patients with diabetes. Targeting epigenetic podocyte changes and associated DNA damage may become a novel therapeutic strategy for preventing progression to end-stage renal disease (ESRD) and provide a possible prognostic marker in diabetic nephropathy. This review summarizes recent advances regarding epigenetic changes, especially DNA methylation, in podocytes in diabetic nephropathy and addresses detection of these alterations in human samples. Additionally, we focused on DNA damage, which is increased under high-glucose conditions and associated with the generation of epigenetic changes in podocytes. Furthermore, epigenetic memory in diabetes is discussed. Understanding the role of epigenetic changes in podocytes in diabetic nephropathy may be of great importance considering the increasing diabetic nephropathy patient population in an aging society.

## Introduction

The worldwide prevalence of type 2 diabetes mellitus has rapidly increased over the past 30 years as a result of changes in the population distribution, namely, aging of the general population (Worldwide trends in diabe, 2016), indicating that the prevalence of microvascular complications, such as diabetic nephropathy (DN) and neuropathy and macrovascular complications, such as cardiovascular diseases, has also increased (Liyanage et al., 2015; Shah and Brownlee, 2016; Yoshida et al., 2020). In particular, DN has been a social issue in that the number of people requiring hemodialysis for end-stage renal disease (ESRD) has been increasing rapidly, and diabetic nephropathy is the most common indication for maintenance hemodialysis (System (2019)., 2019; Jones et al., 2005). However, the mechanisms underlying DN pathogenesis are still not fully understood, and no specific treatment is currently available for DN. Current therapies to manage DN are limited to the control of blood pressure and gluse levels, and treatment with angiotensin-receptor blockers (ARBs) and angiotensin-converting-enzyme (ACE) inhibitors may partially ameliorate proteinuria and delay progression to ESRD (Gregg et al., 2014; Worldwide trends in diabe, 2016).

Classical clinical manifestations of DN include microalbuminuria, which progresses to macroalbuminuria or overt proteinuria over time, and a decline in renal function. Microalbuminuria has been accepted as the earliest marker of DN and damaged kidney glomerular podocytes, which act as a barrier against proteinuria, are a key factor in the prognosis of DN (Jefferson et al., 2008; Hodgin et al., 2015). However, recent epidemiological studies have suggested that microalbuminuria does not always precede renal function loss in diabetes, resulting in a new concept of diabetic kidney disease (DKD) (Kramer et al., 2003; Rigalleau et al., 2007; Kim et al., 2014; Porrini et al., 2015). Distinguishing classical DN from non-albuminuric DKD is difficult in the clinical setting or even from pathological findings, and new prognostic biomarkers other than microalbuminuria are needed.

Recently, growing evidence has shown that epigenetic mechanisms are involved in the initiation and progression of aging, tumorigenesis and lifestyle-related diseases, including diabetes and its complications, such as diabetic nephropathy (Zhou et al., 2012; Reddy and Natarajan, 2015). Combining the results of prior genome-wide association studies (GWAS) and chromatin annotation maps, the importance of transcriptional regulation variants as a mechanism of kidney function has been demonstrated (Pattaro et al., 2016). Moreover, recent epigenome-wide association studies (EWAS) have suggested that the DNA methylation status associated with kidney function can be analyzed using kidney specimens or noninvasive procedures, such as saliva and whole-blood analysis (Sapienza et al., 2011; Ko et al., 2013; Smyth et al., 2014; Wing et al., 2014; Chu et al., 2017).

Based on the importance of podocytes in DKD and disease progression in epigenetic research, this review summarizes recent advances regarding epigenetic changes, especially altered DNA methylation in podocytes, and detection of these alterations in human diabetic nephropathy samples. Additionally, we focused on DNA damage, which is increased under high-glucose conditions and associated with the generation of epigenetic changes in podocytes.

### Podocytes in Diabetic Nephropathy

Kidney podocytes, which are glomerular epithelial cells, are important cells that form a slit membrane and act as a barrier against proteinuria. Podocyte damage causes morphological changes, detachment and apoptosis, leading to proteinuria, glomerulosclerosis and renal failure. The kidney consists of various cell types with different proliferative capacities, and among them, podocytes are distinctive because they are terminally differentiated with a limited ability to proliferate. Accumulating evidence suggests that podocytes may play a pivotal role in kidney aging, especially given that podocyte numbers decrease with age (Hodgin et al., 2015; Nagata, 2016).

The importance of podocyte roles in the pathogenesis of chronic kidney disease (CKD), including DKD, has been clearly recognized by previous studies (Pagtalunan et al., 1997; Diez-Sampedro et al., 2011). Proteinuria is an important clinical diagnostic indicator of DKD, which is correlated with ultrastructural estimation of podocyte foot process effacement (Royal et al., 2020). The association between podocyte foot process effacement and proteinuria has been thoroughly demonstrated in animal model studies (Koop et al., 2003; Jefferson et al., 2008). Long-standing diabetes mellitus can cause diabetic nephropathy with proteinuria as a manifestation of podocytopathy caused by hyperglycemia, impaired insulin receptor signaling, advanced glycation end-product toxicity and glomerular inflammation (Kopp et al., 2020). Furthermore, podocyte-endothelial cell crosstalk regulated by glucocorticoid receptor (GR) plays an important role on renal homeostasis in diabetes (Srivastava et al., 2021a; Srivastava et al., 2021b), suggesting the importance of focusing not only on the podocytes themselves, but also on the interactions between podocytes and other cells in DKD development.

A recent report from the TRIDENT (Transformative Research in Diabetic Nephropathy) cohort highlighted the role of podocyte changes in kidney function decline (Palmer et al., 2021). Podocyte hyperplasia and interstitial fibrosis have been suggested to be significant predictors of ESRD according to a multivariate model. The role of podocyte injury and adaptation in kidney function decline is consistent with a previous report showing the value of segmental sclerosis in predicting kidney function decline in diabetic kidney disease (Mottl et al., 2018). These results indicate that subtypes of podocyte changes in DKD, including podocyte hyperplasia and podocytopathy, may be interesting markers for disease progression as well as targets for podocyte-specific therapeutics.

### DNA Methylation Changes in Podocytes in Diabetic Nephropathy

To understand the mechanisms and consequences of epigenetic changes, the organizational structure of nuclear DNA and chromatin must first be understood. Nuclear DNA is packaged into a histone–protein complex called chromatin. The basic functional unit of chromatin is the nucleosome. Each nucleosome is composed of ∼147-bp double-stranded DNA and a histone octamer containing two copies of each core histone (Kouzarides, 2007; Jones, 2012). Epigenetics is the study of how cells control gene activity without changing the DNA sequence. Epigenetic alterations include DNA methylation, histone modifications, and RNA-based regulation (Lee et al., 2014). In particular, DNA methylation is more stable than other types of epigenetic modifications and may contribute to sustained changes in gene expression. Methylation at the C5 position of cytosine residues in DNA (5-methylcytosine: 5mC) is a major chemical modification in mammalian genomic DNA and several studies have showed the relationships between DNA methylation of cytosine in CpG island and DKD (Bechtel et al., 2010; Tampe et al., 2014). Methylation of cytosine in CpG islands, which are often found in or around the promoter region, usually causes repression of transcription. Cytosine methylation is induced by DNA methyltransferase (DNMT) using S-adenosyl-L-methionine as a methyl group donor. DNMT1 is basically a maintenance methyltransferase that mainly methylates hemimethylated CpG sites during cell division; however, recent studies have revealed that it may also have a role in *de novo* methylation (Fatemi et al., 2001; Vilkaitis et al., 2005; Goyal et al., 2006). DNMT3A is responsible for *de novo* DNA methylation, and DNMT3B is thought to function in *de novo* methylation rather than maintenance methylation. Cooperation between DNMT3 and DNMT1 has been reported to achieve *de novo* DNA methylation more efficiently (Fatemi et al., 2002; Kim et al., 2002). Although the mechanism of DNA methylation in nondividing cells remains unclear, we reported that DNMT1 and DNMT3B have a coordinated role in podocyte DNA methylation (Hishikawa et al., 2019). Interesting results have been reported in neurons, which are also nondividing cells, and both DNMT1 and DNMT3A play important roles in the plasticity of brain function (Feng et al., 2010). Using the conditional knockout mice in nephron progenitor cells, DNMT1 was found to be essential for kidney development, whereas DNMT3A and DNMT3B are dispensable (Li et al., 2019).

**FIGURE 1:**
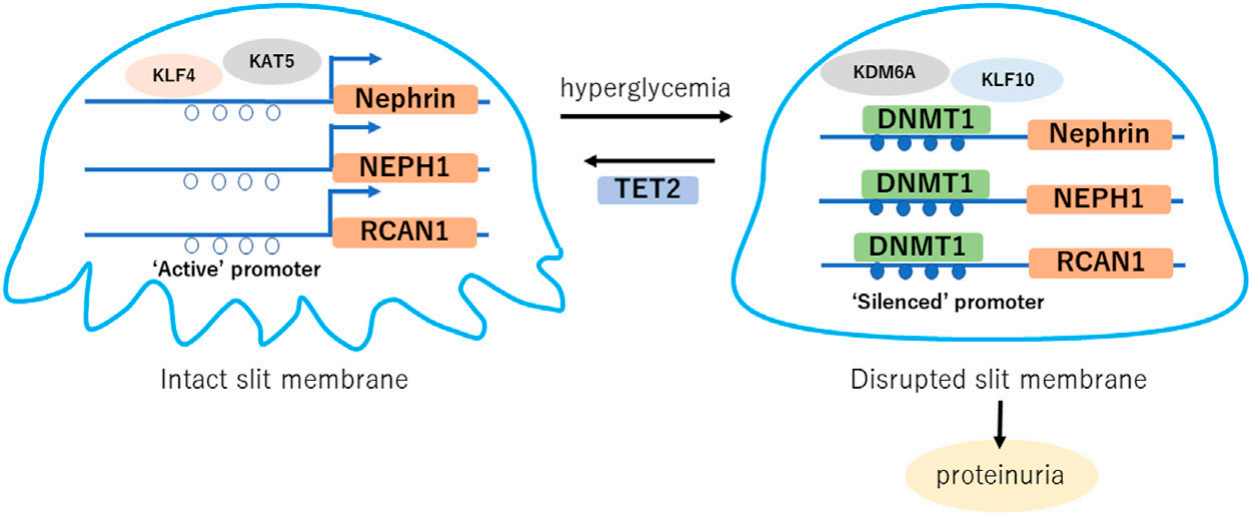
Hyperglycemia induced DNA methylation changes in podocytes. Gene expression such as nephrin, NEPH1 and RCAN1 is maintained with “active” promoter, which forms intact slit diaphragm. Hyperglycemia induces DNA methylation of their promoter regions, which causes “silenced” promoter and decreased expression, leading disruption of the slit membrane. TET2 is a potential factor of podocyte protection through DNA demethylation of the nephrin and NEPH1 promoter regions in diabetes. KLF, Kruppel like factor; KAT5, lysine acetyltransferase 5; NEPH1, Nephrin-like protein 1; KDM6A, lysine demethylase 6A; RCAN1, regulator of calcineurin 1; DNMT1, DNA metyltransfearse 1; TET2, ten-eleven-translocation 2.

Previous reports have suggested the role of DNA methylation changes in podocytes in DKD, as summarized in Figure 1. Decreased expression of transcription factor Kruppel-like Factor 4 (KLF4) in podocytes caused increased DNMT1 binding to the nephrin promoter region, which led to a decrease in nephrin expression. Nephrin is an essential molecule that forms a slit membrane; therefore, decreased nephrin expression induces disruption of the slit membrane and proteinuria (Hayashi et al., 2014; Hayashi et al., 2015). Recently, activation of KDM6A-KLF10 positive feedback loop by hyperglycemia has also been reported to contribute to podocyte dysfunction through decreased nephrin expression by direct binding of KLF10 to the gene promoter together with the recruitment of DNMT1 (Lin et al., 2019). Zhang et al. showed that podocyte DNMT1 may be a promising target for DKD treatment. The decreased DNMT1 expression induced by treatment with 5-azacytidine in DKD model mice recovered nephrin expression and morphological changes in podocytes (Zhang et al., 2017a). Another study also showed that pretreatment with the DNA methyltransferase inhibitor 5-Aza-2′-deoxycytidine alleviates podocyte damage through restoration of suppressed regulator of calcineurin 1 (RCAN1) expression in cultured podocytes (Li et al., 2018).

On the other hand, the demethylation mechanism of 5mC has also been elucidated, which involves an enzyme called ten-eleven-translocation (Tet). Despite fewer reports on the role of Tet in podocytes or kidney diseases than on the role of DNMT, elevated Tet2 is suggested to protective for podocytes (Wan et al., 2021). Further studies are necessary to understand the roles of Tet-induced DNA demethylation in kidney diseases, including DKD.

Recent developments in deep sequencing technology have enabled detection of novel DNA adenine methylation (N^6^-methyladenine, 6mA) in the human genome (Zhu et al., 2018) in addition to 5mC. This modification is mediated by methyltransferase N^6^ adenine–specific DNA methyltransferase 1 (N^6^AMT1) and demethylase alkB homolog 1 (ALKBH1) (Xiao et al., 2018). Related to kidney disease, ALKBH1-mediated DNA N^6^-methyladenine modification triggers vascular calcification in chronic kidney disease (Ouyang et al., 2021). ALKBH family factors mediate demethylation of both DNA and RNA methyladenine, and the 6 mA contents in RNA from the peripheral blood of type 2 diabetic patients and diabetic rats have also been reported to be significantly lower than those in control groups (Shen et al., 2015). Investigation of the roles of 6mA and 5mC may be necessary for a better understanding of the pathogenesis of DKD.

In addition to DNA methylation, numerous reports suggest the importance of histone modifications in diabetic podocytes as shown in previous reviews (Kato and Natarajan, 2019; Dewanjee et al., 2021). In this review, we have focused on DNA methylation in particular, but we would like to refer a few important recent reports on histone modifications. Sirtuin 1 (SIRT1), a nicotinamide adenosine dinucleotide (NAD+)-dependent deacetylase, has been reported to be one of the most important factors involved in the pathogenesis of diabetic nephropathy, which plays a crucial role in regulating histone and DNA methylation through the recruitment of other nuclear enzymes to the chromatin. These epigenetic changes alter podocyte metabolism and function through their morphological changes. For example, decreased SIRT1 in diabetic podocytes caused histone acetylation and upregulation of Claudin-1 gene expression, leading to disruption of the slit membrane and proteinuria (Hasegawa et al., 2013), which could be restored by administration of nicotinamide mononucleotide (NMN) (Yasuda et al., 2021). Recently it is reported that podocyte histone deacetylase (HDAC) 1 and HDAC2 activities were increased in mice podocytopathy models, and podocyte early growth response 1 (EGR1) was increased in proteinuric patients and mice in an HDAC1- and HDAC2-dependent manner (Inoue et al., 2019). Liebisch, M et al. demonstrated a role of histone methylation in diabetic podocytes, showing that advanced glycation end products (AGEs) decreased enhancer of zeste homolog 2 (EZH2) expression in podocytes and consequently reduced H3K27me3 (Liebisch and Wolf, 2020). Majumder et al. showed that histone lysine methyltransferases (HMTs) and histone lysine demethylases (KDMs) regulated H3K27me3 levels at the promoter region of Jag1, which encoded the Notch ligand Jagged1 in podocytes, and were involved in the development of glomerular dysfunction (Majumder et al., 2018). The studies regarding epigenetic changes in podocytes in DN are summarized in Table 1.

**TABLE 1:** Summary of the animal studies regarding epigenetic changes in podocytes in DN.

	Epigenetic changes	Epigenetic modulators	Target molecules	Animal models	References no
1	DNA methylation	KAT5, DNMT1, DNMT3B	Nephrin	BKS.Cg-m+/+Leprdb/J (db/db) mouse model	Hishikawa et al. (2019)
2		KLF4, DNMT1	Nephrin, podocin, vimentin	BALB/c mice or C57BL/6J mice	Hayashi et al. (2014), Hayashi et al. (2015)
3		KDM6A, KLF10, DNMT1	Nephrin, podocin, WT1, synaptopodin	Immortalized mouse podocyte cell line	Lin et al. (2019)
4		DNMT1	Sp1, NFkB, p65	db/db mice	Zhang et al. (2017a)
5		DNMT1	RCAN1	Human podocyte primary cultures and B6/129 mice	Li et al. (2018)
6		TET2	NEPH1, nephrin	Rat and *in vitro* podocyte epithelial-mesenchymal transition (EMT) model	Wan et al. (2021)
7		ALKBH1	N6-methyladenosine	C57BL/6J mice, Oct4F/F (Pou5f1tm1Scho) and Myh11-Cre/ERT2 mice	Ouyang et al. (2021)
8		FTO protein, ALKBH5	N6-methyladenosine	Peripheral blood samples from T2DM patients	Shen et al. (2015)
9		DNMT1	TGF-β1, Rasal1	CD1 mice	Bechtel et al. (2010)
10		TET3	TGF-β1, Rasal1	CD1 mice	Tampe et al. (2014)
11	Histone acetylation	KLF4, acetylated H3K9	Nephrin	BALB/c mice or C57BL/6J mice	Hayashi et al. (2014), Hayashi et al. (2015)
12		SIRT1	Claudin-1	db/db mice	Hasegawa et al. (2013), Yasuda et al. (2021)
13		HDAC1, HDAC2	EGR1	C57BL/6 mice	Inoue et al. (2019)
14	Histone methylation	EZH2, H3K27me3	p27Kip1, RAGE	db/db mice	Liebisch and Wolf, (2020)
			TGF-β1, SNAI1		
15		HMTs, KDMs, H3K27me3	Notch ligand Jagged1	R26Rfl/fl reporter mice	Majumder et al. (2018)

KAT5, lysine acetyltransferase 5; DNMT1 & 3A, DNA methyltransferase 1 & 3A; KLF4, Kruppel-like factor 4; RCAN1, regulator of calcineurin 1; TET2, ten-eleven-translocation 2; ALKBH, α-ketoglutarate-dependent dioxygenase alkB homolog; FTO protein, fat mass-and obesity-associated protein; SIRT1, Surtuin 1; HDAC 1 & 2, histone deacetylase 1 & 2; EFR1, early growth response 1; EZHZ2, enhancer of zeste homolog 2; HMT, histone lysine methyltransferase; KDM, histone lysine demethylase.

### DNA Damage Repair in Podocytes in Diabetic Nephropathy

One of the mechanisms of altered DNA methylation in podocytes without cell division is thought to be involved in the DNA damage repair system. DNA damage is caused by two main sources: exogenous stress, such as UV radiation and chemicals, and endogenous stress, such as reactive oxygen species, stress hormones, DNA replication errors, spontaneous reactions, and mechanical stress (Lieber, 2010; López-Otín et al., 2013). DNA damage can occur in various ways, such as double-strand breaks (DSBs), base adducts, interstrand crosslinks, and mismatches. In particular, DSBs are biologically important because their repair is more difficult than that of other types of DNA damage (Berkovich et al., 2007). When damaged DNA is repaired, epigenetic marks are also reconstituted. Accumulation of repair failures may cause changes in the epigenome, including histone modifications and DNA methylation, and in gene expression (Mortusewicz et al., 2005). DNMT1 is recruited to DSB sites where it colocalizes with phosphorylated histone H2AX (γH2AX) to silence the repaired gene (Mortusewicz et al., 2005). Increased DNA methylation due to DNMT1 action may indicate epigenetic memory denoting former damage (Cuozzo et al., 2007; Orlowski et al., 2011). However, the association of DNA damage repair with aberrant epigenetic states has not been adequately clarified, especially under *in vivo* conditions.

**FIGURE 2:**
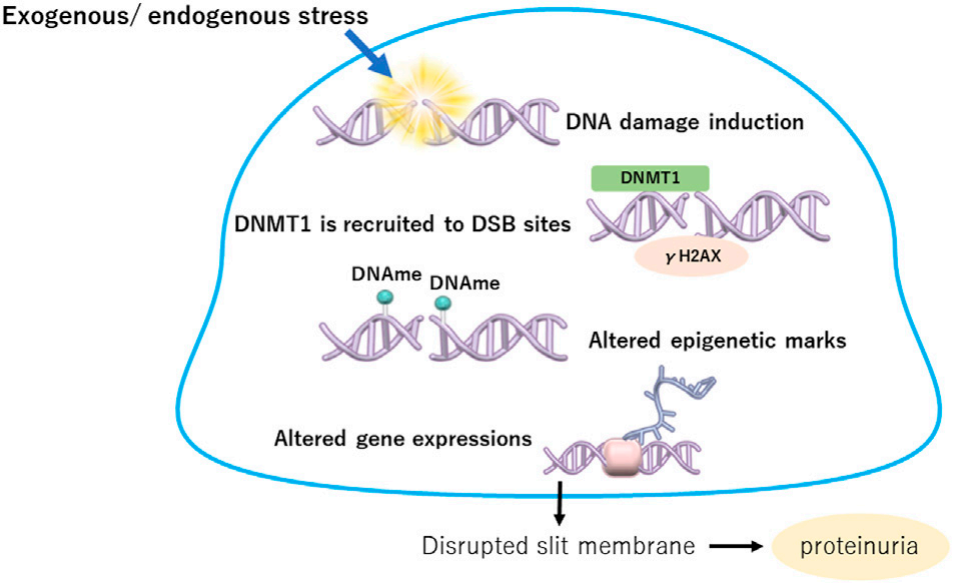
DNA damage repair in podocytes highlighting the epigenetic aspects. Exogenous or endogenous stress causes DNA damage in podocytes. When damaged, DNMT1 is recruited to DSB sites where it colocalizes with phosphorylated histone H2AX (y H2AX) to silence the repaired gene. Increased DNA methylation due to DNMT1 action may indicate epigenetic memory denoting former damage, which induces altered expressions of critical genes in podocytes. DNMT1, DNA metyltransfearse 1; DSB, double strand break; DNAme, DNA methylation.

An estimated 10–50 DSBs per day have been reported in dividing cells (Lieber et al., 2003; Lieber, 2010). Although the number of DSBs induced in podocytes per day is not clear, a large amount of DSB stress may exist around podocytes. Very few DNA DSB sites, which are shown as γH2AX-positive areas, were observed in the kidneys of healthy young mice, suggesting that the DNA repair mechanism may be important in podocytes in physiological states (Hishikawa et al., 2019). Moreover, high-glucose conditions induce genomic and mitochondrial DNA damage in podocytes (Hishikawa et al., 2019; Chen et al., 2020a; Wu et al., 2021). Therefore, protection from DNA damage and promotion of DNA repair in podocytes may be an important treatment strategy for DKD. DNA damage repair in podocytes highlighting the epigenetic aspects is summary in Figure 2.

Recently, we demonstrated the association between DNA damage repair and DNA methylation changes in podocytes in diabetes (Hishikawa et al., 2019). The expression of the DNA repair factor lysine acetyltransferase 5 (KAT5) was reduced in the glomeruli of diabetic nephropathy in mouse models and humans, and knockdown of podocyte KAT5 expression caused focal segmental glomerulosclerosis with increased DNA DSBs and increased DNMT1 and DNMT3B expression with phenotypical changes in podocytes. Although UV radiation caused a significant increase in KAT5 expression in cultured podocytes, high-glucose conditions caused decreased KAT5 expression in podocytes, which led to the accumulation of DNA DSBs in podocytes in diabetic nephropathy.

### Detection of DNA Damage and Epigenetic Changes in Human Samples

Searching for a potential marker predicting the risk for DKD progression may be a promising target for detecting DNA damage and epigenetic changes in human samples. If kidney biopsy is performed, biopsy specimens can be evaluated using various techniques, including not only classical methods such as immunostaining and gene expression analysis but also novel approaches such as proteomics, metabolomics and single-cell RNA-seq analysis (Malone et al., 2020; Satake et al., 2021). Focusing on the assessment of DNA methylation and DNA damage, DNA methylation 5mC and DNA DSBs were evaluated by immunostaining using antibodies against 5mC and γH2AX, respectively. To estimate the amount of DNA DSBs, a previously described long-distance PCR method is also available (Maslov et al., 2013). For example, glomerular DNA methylation evaluated by immunostaining and DNA DSB levels evaluated by immunostaining and the long-distance PCR method were shown to be associated with estimated glomerular filtration rate (eGFR) decline in IgA nephropathy (Hayashi et al., 2020). In the future, recent developments in single-cell epigenomics (Harada et al., 2021) may allow us to analyze altered epigenomes in human kidney samples and to obtain valuable information about kidney disease progression.

Examination of urine samples is easy and less invasive than kidney biopsy, and these samples contain more information than we had previously expected. Urine contains various types of cells, including podocytes. The number of urinary podocytes has received increasing attention in recent years for their usefulness in the diagnosis of early glomerular diseases and in the evaluation of their activity (Fatima et al., 2012). The DNA methylation pattern of proximal tubule-specific loci in urine sediment has been reported to be a potential marker of kidney function decline in diabetes (Marumo et al., 2020). As in tubular epithelial cells, the DNA methylation pattern in urinary podocytes may suggest the kidney prognosis of DKD. Epigenetic patterns in urine-derived cells are quite different among cell types, and detection of epigenomes in specific cell types may be complicated and require ingenuity. Single-cell RNA-seq analysis using urine-derived cells has been reported (Abedini et al., 2021), and single-cell epigenomics in urine may become available in the future.

Altered expression of epigenetic modifiers, such as DNMTs and TETs, can be assessed in whole urine-derived cells, and in our study, DNMT/AQP1 expression was suggested to be significantly correlated with the annual eGFR decline rate after adjustment for age, baseline eGFR, the presence of diabetes and the amount of albuminuria (Hishikawa et al., 2019). Evaluation of podocyte DNA damage using genomic DNA extracted from human urine-derived cells by the quantitative long-distance PCR method may be feasible. Based on the assumption that DNA damage is caused in opened chromatin, DNA damage in the nephrin gene, which is specifically expressed in podocytes, may reflect the level of podocyte DNA damage. Podocyte DNA DSBs were significantly increased in patients with both hypertension and diabetes compared with those in patients with hypertension alone in a study population with an eGFR of approximately 60 ml/min/1.73 m^2^ (Hishikawa et al., 2019).

Detection of DNA methylation changes in whole blood samples has been conducted mainly in DKD patients (Gluck et al., 2019; Park et al., 2019). Interestingly, some altered DNA methylation loci were common to the blood and kidney samples, and some were different. Although the mechanism of DNA methylation changes in blood cells associated with kidney function has not been elucidated, it may be an interesting marker for renal function and prognosis.

### Epigenetic Alterations and Metabolic Memory in Diabetes

Epigenetic regulation is characterized by persistence, as the DNA methylation state is maintained in dividing cells, and by reversibility, as it can be changed by environmental factors and aging. This property suggests that epigenetic changes may be responsible for the “memory effect,” a sustained effect of transient treatment, which is recognized in large clinical trials of diabetes. The landmark Diabetes Control and Complications Trial (DCCT) and the following observational Epidemiology of Diabetes Intervention and Complications (EDIC) study showed that intensive glycemic control at the early stage of Type 1 diabetes delayed the progression of microvascular complications, including nephropathy and neuropathy, compared to conventional therapy, despite similar mean hemoglobin A1c (HbA1c) control at the later stage (de Boer, 2014; Nathan, 2014; Chen et al., 2016). Another study reported similar long-term benefits of intensive glycemic control in patients with type 2 diabetes (Colagiuri et al., 2002; Gaede et al., 2008; Holman et al., 2008). These benefits continued after cessation of the intervention, and the authors called a “legacy effect” of glycemic control.

Recent studies using these DCCT/EDIC cohorts have suggested that DNA methylation patterns in blood cells, especially myeloid cells and hematopoietic stem cells, are significantly associated with HbA1c-associated complications in diabetes (Chen et al., 2016; Chen et al., 2020b). Namely, these studies suggest the possibility that DNA methylation changes in blood cells may substantially mediate the memory effect in diabetes. Whether altered DNA methylation in blood cells is just a marker or has a function and plays a role in disease progression is unclear; however, further study is necessary to elucidate the mechanism combining DNA methylation in blood cells with diabetic complications, including DKD.

Focusing on epigenetic memory in podocytes, Lizotte et al. reported that hyperglycemia induces epigenetic changes in the SHP-1 promoter, including histone modifications, acetylation of H3K9/14 and monomethylation of H3K4, causing its persistent expression and activity and leading to insulin resistance, podocyte dysfunction, and DKD (Carney, 2016; Lizotte et al., 2016). We have previously shown that transcription factor KLF4-mediated epigenetic changes in podocytes are involved in the pathogenesis of CKD, including DKD (Hayashi et al., 2014). Restoration of KLF4 expression in murine podocytes of a diabetic nephropathy model caused a sustained decrease in proteinuria. In addition, the activated renin-angiotensin system (RAS) in CKD induces decreased KLF4 expression, which was recovered by RAS inhibitors (Hayashi et al., 2015). Some clinical studies indicate the memory effect of RAS inhibitors on hypertension (Julius et al., 2006; Sasamura et al., 2013), which is apparent in using high-dose RAS inhibitors in animal models (Nakaya et al., 2001; Ishiguro et al., 2007; Ishiguro et al., 2009; Hayashi et al., 2010). Further clinical studies are necessary to evaluate the memory effect of RAS inhibitors on DKD.

## Discussion

Based on a number of reports, epigenetic mechanisms undoubtedly play an important role in the pathogenesis of diabetic nephropathy. Podocytes, which are one of the key factors influencing kidney disease progression in diabetes, are terminally differentiated cells with limited proliferation ability and thus are expected to be prone to accumulate DNA damage and epigenetic changes.

The significance of focusing on epigenetic changes is that they are likely to be maintained in a manner that accompanies the genome, which may have a sustained effect called “metabolic memory” in diabetes. Therefore, epigenome-targeted therapy may be capable of ameliorating the sustained effects of hyperglycemia in diabetes and its complications. Although DNA methyltransferase inhibitors or histone deacetylation (HDAC) inhibitors have already been used in patients with hematological malignancies, applying these drugs directly to DKD is difficult. The epigenetic state is responsible for the characteristics of individual cells and tissues, and since it differs depending on cell types, treatment with uniform epigenome-modifying drugs is fraught with concerns about side effects. Targeting tissue-specific pathways contributing to tissue-specific epigenetic changes is desirable. The DNA damage repair pathway is highly cell-specific, so targeting DNA damage repair related to epigenetic changes may be possible to achieve more specific epigenome-targeted therapy. Another approach is an investigation of delivery systems to specific cell types, including podocytes. The development of a podocyte-specific delivery system could make it possible to modulate the podocyte epigenome directory. Recently, several clinical trials of gene therapy using the genome editing technique of the CRISPR–Cas9 system have been reported (Reddy et al., 2015; Gillmore et al., 2021; Schmidt et al., 2021). Currently, epigenome editing with CRISPR-based systems has been investigated *in vivo* (Gemberling et al., 2021), and the technology was used to treat mouse models of diabetes, muscular dystrophy, and acute kidney disease (Liao et al., 2017). Future therapeutic applications of epigenome editing for DKD in humans are expected.

Investigation of DNA damage and altered DNA methylation of the kidney may contribute to the development of a marker for estimating the renal prognosis of DKD as well as a therapeutic target. In particular, recent technical advances have made us aware of the potential of urine samples. Urine-derived renal progenitor cells are available (Rahman et al., 2020), and urine-derived induced pluripotent stem (iPS) cells are used in drug discovery and toxicology, as well as in regenerative medicine (Bento et al., 2020). Evaluation of DNA damage in specific genes in the kidney using urine-derived cells may indicate site-specific renal damage without kidney biopsy. Further study to clarify the association of renal histological changes with the DNA damage level of urine-derived cells is needed.

## Future Directions

Finally in this section, future directions of possible therapeutics for DN are summarized. Clinically, the current general managements in DKD include strict metabolic controls of glycemia, blood pressure, body weight, and lipids, augmenting the efficacy of the renin-angiotensin system (RAS) blockade to ameliorate proteinuria (Worldwide Trends in Diabe, 2016). Few effective drugs had been existed to hinder DKD progression, however, sodium-glucose cotransporter 2 inhibitor (SGLT2i) has recently been expected to suppress DKD progression (Wanner et al., 2016; Neal et al., 2017; Wiviott et al., 2019). Until now, candidates of therapeutic agents for protection of diabetic podocytes have been reported in mouse models, including DPP-4 inhibitor for antioxidative and antifibrotic effects (Takashima et al., 2016; Spencer et al., 2018), statins for maintaining slit diaphragm proteins (Tonolo et al., 2006), SIRT3 for inhibition of mitochondrial oxidative stress (Locatelli et al., 2020), JAK-STAT inhibitors for appropriate autophagy (Zhang et al., 2017b; Chen et al., 2021), ACE2 for maintaining nephrin expression and decreased transforming growth factor-β1 (Nadarajah et al., 2012) and peptide N-acetyl-seryl-aspartyl-lysyl-proline (AcSDKP) as an endogenous antifibrotic mediator (Srivastava et al., 2020). We believe that therapies targeting podocyte epigenome, which is the theme of this review, could also be a hopeful candidate for future therapies for DKD. These new candidates for therapeutic targets may produce additive or even synergistic effects when used in combination with currently used RAS blockers and SGLT2i, and further clinical studies are expected.

In the future, we may witness an era in which the genomic and epigenomic information of blood and urine cells is comprehensively analyzed in each patient, and then therapeutic strategies are formulated based on the information. Basic and clinical studies are needed to clarify the role of epigenetic changes in DKD progression. Moreover, such detailed information must be integrated to ultimately understand the pathologies occurring within each patient with DKD.
